# Mechanism of HMGB1–RAGE in Kawasaki disease with coronary artery injury

**DOI:** 10.1186/s40001-020-00406-5

**Published:** 2020-03-17

**Authors:** Biying Qian, Hua Huang, Mingye Cheng, Tingting Qin, Tao Chen, Jianmei Zhao

**Affiliations:** 1 Department of Paediatrics, Affiliated Hospital of Nantong University, Nantong, 226001 Jiangsu People’s Republic of China; 2 Medical College of Nantong university, Nantong, 226001 Jiangsu People’s Republic of China; 3 Department of Emergency Medicine, Shanghai Children’s Hospital, Shanghai Jiaotong University, Shanghai, 200062 People’s Republic of China

**Keywords:** Kawasaki disease, Coronary artery lesion, HMGB1, RAGE

## Abstract

**Background:**

Kawasaki disease (KD) is a common, yet unknown etiology disease in Asian countries, which causes acquired heart disease in childhood. It is characterized by an inflammatory acute febrile vasculitis of medium-sized arteries, particularly the coronary arteries. High-mobility group box-1 protein (HMGB1) is a non-histone chromosomal-binding protein present in the nucleus of eukaryotic cells, which contains 215 amino acid residues. Although the cellular signal transduction mechanisms of HMGB1 are currently unclear, the important role of the receptor for advanced glycation end-products (RAGE), the main receptor for HMGB1 has been reported in detail. The purpose of our study was to verify the mechanism and clinical significance of HMGB1-RAGE in coronary artery injury of Kawasaki disease.

**Methods:**

52 blood samples of patients in KD were collected, and the coronary artery *Z* score was calculated according to the echocardiographic results. The *Z* score ≥ 2.0 was classified as coronary artery lesions (CAL); otherwise, it was no-coronary artery lesions (NCAL). In addition, the fever group and control group were set. Among them, the fever group were children with fever due to respiratory tract infection at the same time period as KD (heat peak ≥ 38.5 ℃). The normal group were children at a routine physical examination in the outpatient clinic of Nantong University and the physical examination center of the child care insurance, and there were no infectious diseases and heart diseases. The serum levels of HMGB1, RAGE, and NF-κB in each group were detected by ELISA. The animal model of KD was established using the New Zealand young rabbits. We used RT-qPCR/H&E staining/immunohistochemistry/ELISA and western blot to detect the level of HMGB1/RAGE and NF-κB.

**Results:**

In this study, we found that the HMGB1/RAGE/NF-κB axis was elevated in the serum of children with KD. In addition, an animal model of KD was subsequently prepared to examine the pathological changes of the coronary arteries. We found that the serum levels of HMGB1/RAGE/NF-κB in rabbits with KD were significantly higher than those of the control group. Moreover, the lumen diameter of the coronary artery was slightly enlarged, and the wall of the tube became thinner and deformed. In addition, the HMGB1/RAGE/NF-κB levels in the coronary artery were higher in the rabbits with KD in the acute phase.

**Conclusions:**

On the whole, the findings of this study demonstrate that the expression of HMGB1/RAGE/NF-κB is altered at different stages of KD, suggesting that the HMGB1/RAGE/NF-κB signaling pathway plays an important role in vascular injury in KD. The results of this study may have important implications for the early warning of coronary artery lesions in KD.

## Background

Kawasaki disease (KD) is an autoimmune systemic vasculitis disease, which is common among children. It is self-limiting and often occurs in children under 5 years of age [1]. It can involve small and medium blood vessels, particularly the coronary arteries, and can lead to coronary dilatation, stenosis, and aneurysms, as well as myocardial infarction [2]. The incidence of coronary artery lesions (CALs) is 25–30% in children without regular treatment, while it will be reduced to 5% with the correct treatment during the early period [3]. In developed countries, CALs caused by KD have replaced rheumatic fever as the most common acquired heart disease among children, and may become one of the risk factors for ischemic heart disease in adulthood. At autopsy, the coronary artery has been found to be the most damaging site. Other sites, include the aorta, abdominal aorta, carotid artery, subclavian artery, and pulmonary artery [4]. The pathological changes are similar to those of infant nodular polyarteritis, with arterial full-thickness inflammation, intimal thickening, granulocyte and monocyte infiltration, internal elastic layer and medial membrane rupture, vascular wall necrosis, and aneurysm formation. However, with the increasing number of new medications being developed, and the use of intravenous immunoglobulin (IVIG) therapy, the mortality rate of patients with KD has markedly decreased, and thus the amount of corpses available for autopsy has also reduced. Thus, it is rare for scientists to be able to obtain a coronary artery for the use in further research [5]. Therefore, in this study, a rabbit model of KD was established, and the pathogenesis of KD was further explored at the histological and molecular levels.

High-mobility group box-1 protein (HMGB1) belongs to the HMG family, and is a highly conserved non-histone DNA-binding protein which is widely present in the eukaryotic cell [6, 7]. When the cells in the body are damaged or become necrotic, HMGB1 is passively released into the nucleus. Biologically activated HMGB1 promotes immune cells, including various cytokines (such as TNF-α and IL-1) and chemokines, maturation, and migration. Following the synthesis and release of HMGB1, pro-inflammatory factors, such as TNF-α and IL-1 further promote mononuclear/macrophages, endothelial cells, neuronal cells, NK cells, and other cells to actively secrete HMGB1; thus, forming a positive feedback loop. This leads to the continuous promotion of the inflammatory response [6, 8, 9].

The receptor for advanced glycation end products (RAGE) is a high-affinity receptor of HMGB1 currently known, while not the only receptor for HMGB1 [10]. RAGE is expressed in various types of cells, such as smooth muscle cells, endothelial cells, nerve cells, monocytes/macrophages, and others [11]. Under normal conditions, RAGE is expressed at low levels on the cell surface. However, when its ligand molecule increases, the expression of RAGE is up-regulated. The binding of HMGB1 to RAGE promotes the activation of mitogen, and the phosphorylation of protein kinases (such as p38 kinase, SAPK/JNK, and ERK1/2), and subsequently activates various signaling pathways, such as NF-κB and MAPKp38, inducing the production of inflammatory cytokines and chemokines [12]. Moreover, it is involved in the process of immune cell maturation, axon growth, and tumor proliferation [13]. To date, approximately 6 isoforms including the full-length transmembrane receptor have been found and 5 of these 6 isoforms lack the transmembrane domain, and are thus believed to be secreted from cells. Generally, these isoforms are referred to as soluble RAGE (sRAGE) or endogenous secretory RAGE (esRAGE) [14, 15].

The serum levels of HMGB1 have been shown to be significantly increased in the acute phase of KD [16]. A previous study demonstrated that in the acute phase of KD, the serum levels of HMGB1are higher than those observed upon recovery [17]. In addition, in the acute phase of KD, HMGB1 is involved in the inflammatory reaction and coronary artery lesions become more heavily calcified; however, the exact mechanisms responsible for this injury remain unclear.

In this pilot study, we examined the hypothesis that the expression of HMGB1/RAGE/NF-κB in children with KD and in a rabbit model of KD could be altered. This change could be observed in CALs caused by KD. Moreover, the pathological mechanisms of coronary artery injury in KD were further investigated. For this purpose, serum from children with KD was collected at the acute phase and recovery phase at first. Subsequently, a rabbit model of KD was constructed. We further aimed to prove our hypothesis through research experiments, such as hematoxylin–eosin staining, immunohistochemistry, western blot analysis, ELISA, etc.

## Materials and methods

### Z score

The Z score uses body surface area as the normalized dimension (mean standard deviation) and standardized regression analysis based on continuous measurements, allowing comparison across time and population [18]. ① The inside diameter of those with no dilatation of the coronary arteries was always less than 2.0; ② only during the expansion, the *Z* score was 2.0 to 2.5, or the first *Z* score was less than 2.0, and the *Z* score decreased by more than 1 at follow-up review; ③ small tumor 2.5 < * Z* score < 5; ④ medium tumor 5 ≤ *Z* score < 10, and the absolute value of the inner diameter is < 8 mm; ⑤The value of large or large tumors is ≥ 10, or the absolute value of the inner diameter is > 8 mm [19]. At present, coronary *Z* score obtained by echocardiography has become the main follow-up method for children with KD. There are many formulas currently for calculating the *Z* score, such as in the United States, Japan, Singapore, and Canada. The following two *Z* score calculation sites are provided from https://raise.umin.jp/zsp/calculator/#sub from the Scientific Committee of the Kawasaki Disease Society of Japan and https://zscore.chboston. org/ from the Heart Center of Boston Children's Hospital in the United States.[20].

We used a database recommended by Japan. According to the *Z* score (≥ 2.0), we determined the presence of a coronary artery lesion, and the children in acute phase of KD were divided into the CAL group and NCAL group.

### Patient specimen of KD

All patients diagnosed with KD had fever for at least 5 days and met at least 4 of the 5 clinical criteria for KD (rash, conjunctival injection, cervical lymphadenopathy, oral mucosal changes, and changes in the extremities), or 3 of the 5 criteria along with coronary artery abnormalities documented by an echocardiogram [21]. Patients diagnosed with acute upper respiratory infection and herpangina were prospectively enrolled as febrile control subjects. In this study, the serum of 52 children with acute KD, 14 healthy controls, 14 febrile children, 44 convalescent KD children, and 44 children with subacute KD was collected from The Affiliated Hospital of Nantong University (Nantong, China) between April, 2018 and December, 2018 (Table 1). The investigation conformed with the principles outlined in the Declaration of Helsinki. The collected specimens were centrifuged to separate the serum, and then stored in −80˚C until use. The study protocol was approved by the Institution Review Board of The Affiliated Hospital of Nantong University. Informed consent was obtained from the parents.

**Table 1:** Characteristics of the clinic index in different groups

Groups	Normal	Febrile	Convalescent KD	Subacute KD	Acute KD	
					CAL	NCAL
Number	14	14	44	44	10	42
Male	6 (42.9%)	7 (50%)	28 (63.6%)	26 (59.1%)	6 (60%)	25 (59.5%)
Female	8 (57.1%)	7 (50%)	16 (36.4%)	18 (40.9%)	4 (30%)	17 (40.5%)
Age (months)	22.09 ± 19.08	40.45 ± 20.63	31.27 ± 24,84	28.37 ± 21,63	29.0 ± 22.36	25.42 ± 25.23
WBC (× 10^9^/L)	4.34 ± 3.01	10.52 ± 6.25	7.24 ± 4.25	10.73 ± 4.97	12.13 ± 5.37	13.26 ± 6.02
PLT (× 10^9^/L)	282.05 ± 103.61	304.42 ± 95.73	574.19 ± 152.81	458.52 ± 187.63	389.00 ± 74.94	370.68 ± 146.65
Z score	–	–	–	–	2.27 ± 0.19	0.14 ± 0.54

The values were expressed as the means ± SD. The *Z* scores were calculated by the website of https://raise.umin.jp/zsp/calculator/

*KD* Kawasaki disease, *WBC* white blood cell, *PLT* platelet

### Preparation of animal models, animal sacrificing and materials

The Laboratory Animal Ethics Committee of Nantong University approved the design of this study. The care and handling of rabbits followed the guidelines for the Administration of Experimental Animals of Jiangsu province. Eighteen male and female weanling New Zealand rabbits at 4–5 weeks of age and each weighing 500–1,000 g were purchased from Jiangsu Zhenlin Biotechnology Co., Ltd. [License No.: SCXK (Su) 2016–0008]. The rabbits were randomly and equally divided into 3 groups as follows: The normal group (N), the severe group (S), and the recovery group (R). The rabbits in group S and R received an intravenous (i.v.) injection twice into the marginal ear vein of 3.0 ml/kg BSA (10%, Shanghai Shenggong Bioengineering Co., Ltd., batch number AD0023) respectively, at 14-day intervals [22]. The animals in group N received 3.0 ml/kg saline in the same time with group S and R. On the 10th day after the establishment of the model (acute phase), 5 ml of arterial/venous blood of group N and S was acquired from the heart, and the rabbits in group S were anesthetized with pentobarbital, and the heart was rapidly removed. The coronary artery was picked out under a microscope and removed. The left anterior descending artery was fixed with 4% paraformaldehyde, and other coronary artery branches were stored at −80 ℃. On the 28th day of the model (recovery period), blood was collected from the hearts of the remaining rabbits in groups N and R, and coronary artery specimens were acquired in the same manner.

### Hematoxylin–eosin (H&E) staining and immunohistochemical staining

The left anterior descending artery was fixed with 4% paraformaldehyde for 24 h, then dehydrated by 20% sucrose solution for 24 h, and 30% sucrose was allowed to sink for 24 h. The tissue was embedded with an OCT frozen section embedding agent (Sakura, stock no. 4583) and sliced on a cryostat (Leica CM1900 UV) to a thickness of 6 μm. This was followed by H&E staining, alcohol dehydration, xylene transparentizion, and neutral gum sealing. For immunohistochemical staining, the sections were washed with 0.01 mol/l phosphate buffer for 5 min, 3 times, and the citrate buffer was autoclaved for antigen retrieval. The immunohistochemical two-step test kit (Nakasu Jinqiao, item no. PV-9000) was used in this experiment. First, the endogenous peroxidase blocker was applied for 20 min, then blocked with goat serum for 30 min, and the antibody was added in a dropwise manner overnight (Abcam, ab49849, 1:1,000). After the second day of rewarming, the reaction enhancer fluid was added for 20 min to enhance antigen–antibody combination. The enzyme-labeled goat anti-mouse/rabbit IgG polymer was then added for 20 min, plus DAB color developing agent for 10 s, and the nuclei were counterstained with hematoxylin for 10 s. Finally, alcohol gradient was used for dehydration, xylene for transparentizion, and neutral balsam for sealing. This was followed by observation under an optical microscope and filming. Coronary artery tissue was analyzed using ImageJ software, and the positive expression absorbance values of brown-yellow particles on the coronary arteries were counted.

### Total RNA isolation and reverse transcription-quantitative polymerase chain reaction (RT-qPCR)

A total of 2 ml of whole blood was immediately injected into heparin anticoagulation tubes, and lymphocyte separation solution was added (Institute of Hematology, Chinese Academy of Medical Sciences); the peripheral blood mononuclear cells (PBMCs) were isolated by density gradient centrifugation. Total RNA was isolated using Trizol reagent (Shanghai Shenggong Bioengineering Co., Ltd.). The concentration and purity of the extracted RNA were measured spectrophotometrically at A260 and A280. Total RNA 2 μg was reverse transcribed into cDNA was using the RevertAid First Strand cDNA Synthesis Kit. According to the sequence of GenBank, primers were designed using Primer Premier 5.0 and synthesized by Shanghai Bioengineering Technology Service Co., Ltd. PCR amplification was performed using SYBR-Green master mix and real-time PCR instrumentation using RT-PCR. All real-time PCRs were performed at least in triplicate. Primer sequence of HMGB1, RAGE and NF-κB are as follows: HMGB1 forward, AGCCGAGAGGCAAAATGTCA and reverse, AGTTGACGGAAGCATCTAGG; RAGE forward, CATCTACTCCAAGCAGCCGTACAC and reverse, GGCACTCCACACGTCCATCTTG; NF-κB forward, TGCCTCTGTCGCCGTCTTCC and reverse, CGTGAAGCTGAGTCTGCGGAATG; and β-actin forward, GCAAGTGCTTCTAGGCGGACTG and reverse, CTGCTGTCACCTTCACCGTTCC.

### Enzyme-linked immunosorbent assay (ELISA)

The serum level of HMGB1/RAGE and NF-κB in children and the rabbits in the model of KD were measured using ELISA kits (Shanghai Enzyme-linked Biotechnology Co., Ltd.) according to the manufacturer’s instructions. RAGE measured in serum is referred to sRAGE.

### Western blot analysis

Protein extracts from the coronary artery were separated by SDS-PAGE (11–15% acrylamide). After being transferred to PVDF membranes and blocking for 2 h, primary antibody was added overnight, followed by washing in PBS, application of a secondary HRP-conjugated antibody and development using an ECL system (Absin Bioscience). Rabbit anti-HMGB1 (1:1,000; Abcam, ab18256), goat anti-RAGE (1:1,000; RD systems, AF1145), rabbit anti-NF-κB (1:1,000; Cell Signaling Technology, 8242) mouse anti-*β*-actin (1:1,000; Proteintech, 60,008-1-Ig) were used as primary antibodies. All western blots in this study were subjected to different exposures: From 10 s to 10 min, and the optimal exposures were selected for data presentation.

### Statistical analysis

Histological images were acquired with an Olympus BX51 microscope with objective lenses of Plan Apo at ×10, ×20, and ×40 magnifications and with a color CCD camera (DFC320, Leica) for digital micrographs. For the quantification of positive values, digital micrographs of brown granules of specimens stained by DAB were processed by binarization of the images with ImageJ software. Quantitative data are presented in bar graphs. All investigated variables were normally distributed. Statistical analyses were conducted with SPSS software. Comparisons between 2 groups were carried out using t test, and those among multiple groups were carried out using Tukey's honestly significant difference analysis. For all analyses, *P* < 0.05 was considered to indicate a statistically significant difference.

## Results

### Expression of HMGB1/sRAGE/NF-κB in KD children

**Fig. 1:**
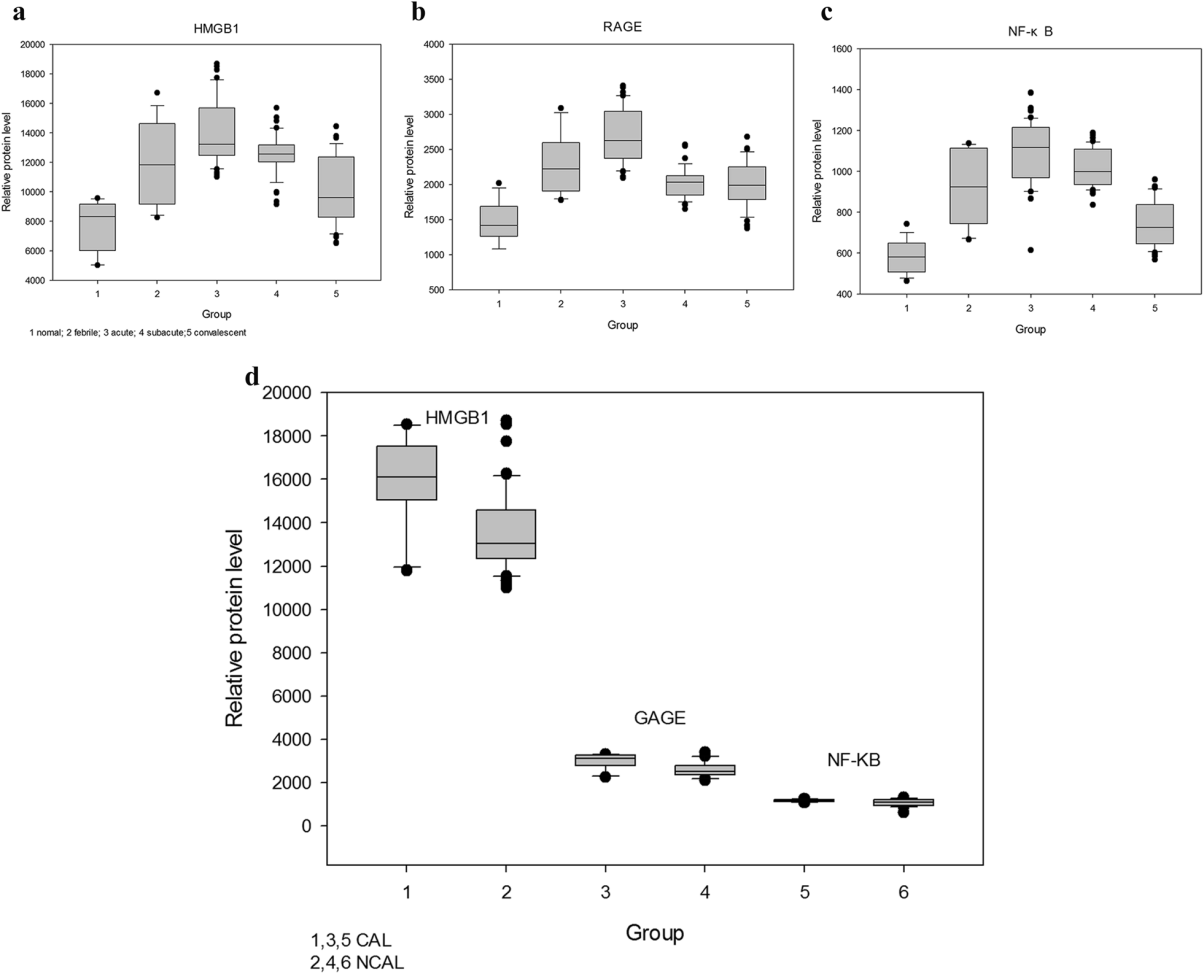
HMGB1/RAGE/NF-κB proteins detected by ELISA in normal children, children with febrile, children with KD in acute phase, children with KD in subacute phase, and children with KD in convalescent phase. *P*<0.05 compared with “normal group”, *P*<0.05 compared with “convalescent phase” (**a**–**c**). The expression levels of HMGB1/RAGE/NF-κB in CAL group and NCAL group in acute phase. *P*<0.05 compared with “NCAL group” (**d**)

ELISA was used to detect the expression levels of HMGB1/sRAGE/NF-κB in children with KD. We found that the expression of the above-mentioned indicators markedly increased in the acute phase of KD. As the disease recovered, the index gradually decreased in the subacute phase and recovery phase. The results revealed that the levels of the above-mentioned indicators were significantly higher in the CAL group, and the difference was statistically significant compared with the NCAL group (*P* < 0.05) (Fig. 1).

We then established a young rabbit model of KD to further examine the association between HMGB1-RAGE with KD and KD CAL.

### Histological changes in the coronary arteries of the rabbit model for KD

#### H&E staining

**Fig. 2:**
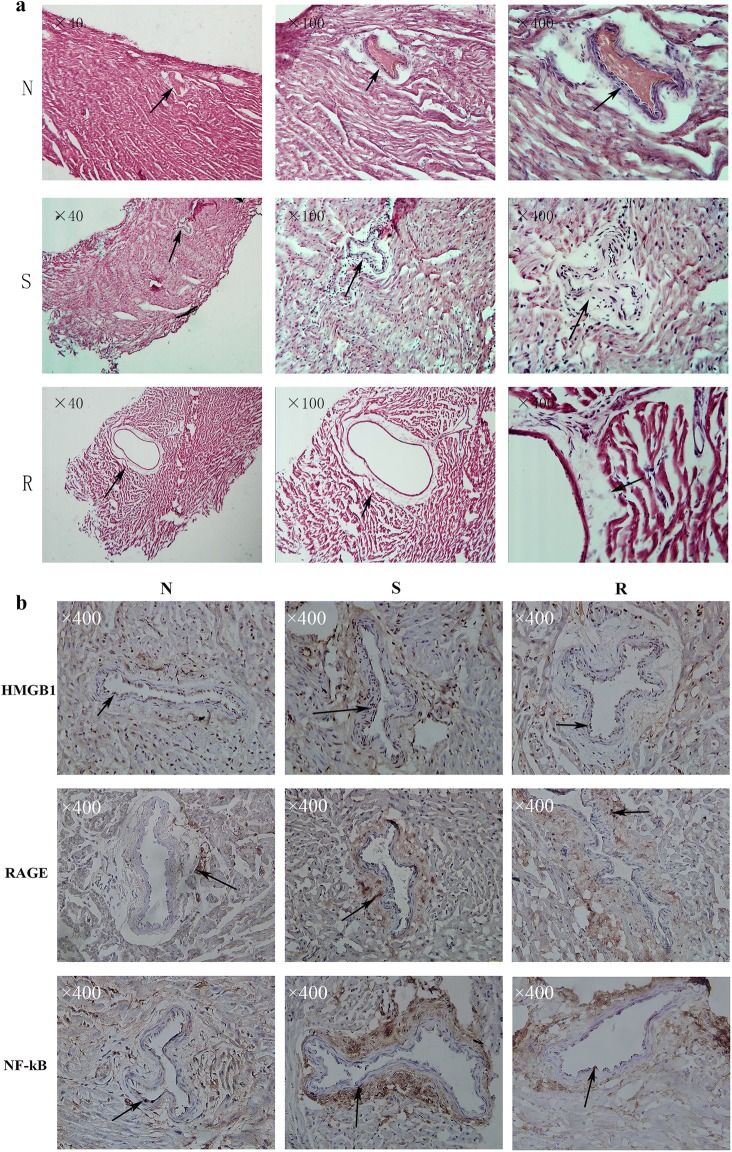
The coronary artery structure in group N was clear, the intima was evident, the lumen size was moderate, and the thickness was uniform; however, in group S, the lumen was slightly enlarged, the wall of the tube became thinner, and the middle layer was more evident. In the group R, the coronary artery became larger and the wall became thinner and deformed. The arrow points to the coronary artery. The picture showed the results in three kinds of magnification multiple: ×100, ×200, ×400 (**a**). As indicated by the arrow, the brown-yellow particles can be seen in the endothelial cells and in the smooth muscle cells. And HMGB1/RAGE/NF-κB levels were highly expressed in the group S analyzed by ImageJ (×400) (**b**)

The results revealed that the coronary artery structure of the normal group (group N) was clear, the intima was evident, the lumen size was moderate, and the thickness was uniform; however, in the coronary artery of the acute phase group (group S), the lumen was slightly enlarged, the wall of the tube became thinner, and the middle layer was more evident. In the recovery group (group R), the coronary artery became larger and the wall became thinner and deformed. In short, the coronary artery of the S group has begun to change, but not as obvious as the R group (Fig. 2a).

#### Immunohistochemical staining

The main expression of brown-yellow particles in the intima endothelial cells was observed. HMGB1 was mainly expressed in the nucleus of endothelial cells, and was also expressed at low levels in smooth muscle cells. RAGE and NF-κB were expressed in both the nucleus and cytoplasm (Fig. 2b). Positive expression levels of HMGB1 in group N, group S, and group R were 5.21 ± 1.35, 14.55 ± 2.01 and 6.24 ± 1.17, respectively; those of RAGE were 6.75 ± 1.27, 13.15 ± 1.64 and 8.37 ± 0.77, and those of NF-κB were 6.19 ± 0.89, 11.81 ± 1.44 and 6.59 ± 0.84, respectively. The levels in group S were higher than those in group N and group R, and those in group R were slightly higher than those in group N. The difference was statistically significant (*P* < 0.05).

### Changes in mRNA expression in the PBMCs rabbit model of KD

**Fig. 3:**
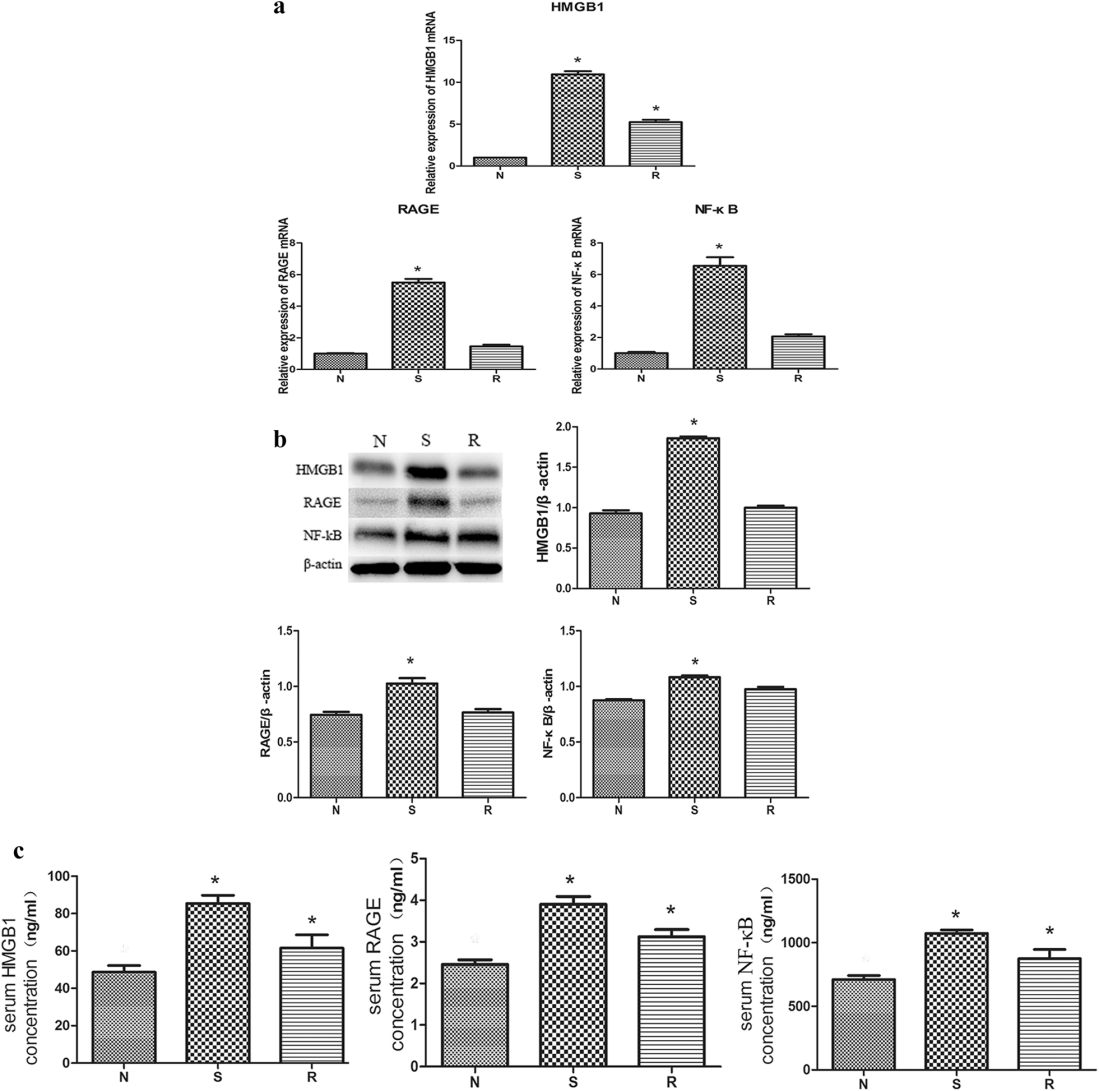
Relative expression levels of HMGB1/RAGE/NF-κB mRNA detected by RT-qPCR. The HMGB1/RAGE/NF-κB mRNA levels were highly expressed in group S than in group N (**a**). The expression levels of HMGB1/RAGE/NF-κB proteins in coronary artery of rabbit model with KD detected by western blot analysis. The levels in group S are higher compared with group N (**b**). The expression levels of HMGB1/RAGE/NF-κB proteins in the serum of rabbit model with KD detected by ELISA, those have high expression in both group S and group R compared with group N (**c**). * show that *P*<0.05 compared with “group N”. *N* normal group, *S* severe group, *R* recovery group

For the rabbit model of KD, the levels of HMGB1/RAGE/NF-κB mRNA in PBMCs of groups N/S/R were as follows: HMGB1: 1.0 ± 0.01, 10.95 ± 0.65 and 5.25 ± 0.47; RAGE: 1.00 ± 0.06, 5.50 ± 0.39 and 1.47 ± 0.18; NF-κB: 1.00 ± 0.13, 6.55 ± 0.95 and 2.07 ± 0.23. The values in group S were significantly higher than those in group N and group R with statistical significance (*P* < 0.05). For HMGB1, the values in group R were slightly higher than those in group N. The difference was statistically significant (*P* < 0.05). For RAGE and NF-κB, the values in group R were slightly higher than those in the normal group, although the difference was not statistically significant (*P* > 0.05) (Fig. 3a).

### Protein expression relative levels in the rabbit model of KD

#### Western blot analysis

Western blot analysis revealed that the HMGB1/RAGE/NF-κB protein level was up-regulated in group S in the young rabbit model of KD, compared with group R and group N, with statistical significance (*P* < 0.05). The levels in the recovery group were higher than those in the normal group, and the difference was statistically significant (*P* < 0.05) (Fig. 3b).

#### ELISA

Subsequent ELISA revealed that the protein levels of HMGB1/sRAGE/NF-κB in group S were significantly higher than those in group N and R (*P* < 0.05). In group R, the levels of HMGB1/sRAGE/NF-κB were still higher than those of group N, although there was no significant difference compared with group N (*P* > 0.05) (Fig. 3c).

## Discussion

Kawasaki disease is a juvenile idiopathic immune disease that is the leading cause of childhood acquired heart disease [23]. Once a coronary artery lesion is formed, which is known as a coronary aneurysm, it may cause lifelong harm to the child and may even cause sudden death. Many researchers have proven that the early detection of correct treatment can prevent coronary aneurysms. High-mobility group box-1 (HMGB1), also known as amphoteric protein, is a type of non-group DNA-binding protein that is widely distributed in higher eukaryotes, and is secreted outside the cell following tissue damage or stimulation by pathogens, which induces different inflammatory processes [7]. HMGB1 needs to bind to receptors on the surface of target cell membranes, and can then be synthesized and released by signal transduction systems, such as mitogen-activated protein kinases (MAPK) and nuclear factor (NF)-κB. Currently, the receptor for advanced glycation end products (RAGE), Toll-like receptor 2 (TLR2), and Toll-like receptor 4 (TLR4) have been found [24]. RAGE belongs to the immunoglobulin superfamily transmembrane receptor, and is expressed in endothelial cells, smooth muscle cells, neuronal cells, monocyte macrophages, and various stem cells [25]. After binding to HMGB1, RAGE mediates multiple intracellular signaling pathways, including NF-κB, JAK/STAT, and MAPK families, and exerts a wide range of physiological and pathological effects [26]. HMGB1 levels have been shown to be higher in 27 children by KD than in healthy controls, as measured by Hoshina [27–29].

In order to be consistent with these studies, we collected serum samples from children in different phases of KD. Due to the current timely treatment of Kawasaki disease, the incidence of coronary artery injury is greatly reduced, so only 10 CAL cases were collected in this study. We found that the expression of HMGB1/sRAGE/NF-κB increased in the acute phase of KD. Notably, in the acute phase of KD, the CAL group exhibited higher levels than the NCAL group, as regards these three indicators [30]. Furthermore, we established a rabbit model of KD. By observing the pathological sections of the coronary artery at different stages, we found that the coronary artery of the rabbit model of KD exhibited luminal dilatation, and the wall had become thinner and deformed. The pathological changes in the coronary arteries in rabbits are similar to those in children with KD. We further found that HMGB1, RAGE, and NF-κB were located in the endothelial cells of the coronary arteries, in which HMGB1 was mainly expressed in the nucleus, and RAGE and NF-κB are in both the nucleus and cytoplasm. By detecting the expression of HMGB1, RAGE, and NF-κB in coronary endothelial cells, we found that the levels of the three indicators were increased in the S group, and in PBMCs that the mRNA expression of HMGB1, RAGE, and NF-κB were also increased. By detecting the expression of HMGB1, sRAGE, and NF-κB in serum, we found that the levels of the three indicators were increased in the S group. Although there are reports that sRAGE is reduced in the acute phase of KD [31], we tested the serum of patients with KD and in a young rabbit model of KD, and found that it was increased in the acute phase, which may be related to the number of cases or time or regional disparity. In addition, some studies have reported that sRAGE is elevated in the acute phase of some diseases; for example, in patients with infection-related ARDS, sRAGE levels have been shown to be much higher than those in the control subjects [32]. In summary, we hypothesized that HMGB1-RAGE may be associated with the development of KD; particularly, in the acute phase when HMGB1 accumulates in coronary endothelial cells, causing coronary vasculitis and promoting coronary artery dilatation. We aim to carry out further research to confirm these results. In summary, HMGB1 can be used as an indicator of inflammation in KD, and has a high clinical value in suggesting coronary artery injury. Therefore, the clinical treatment of KD with active anti-inflammatory, anticoagulant, high-dose gamma globulin infusion, etc., can also block the secretion of HMGB1 at an early stage to prevent coronary artery injury and reduce the occurrence of coronary aneurysms. Limitations of this study: The effect of HMGB1 inhibitors on KD young rabbits has not been verified from animal experiments.

## Conclusion

The findings of this study indicate that the expression of HMGB1, RAGE, and NF-κB were significantly increased in the acute phase of children with KD, which was of great value for the early diagnosis of KD, and the expression of HMGB1, RAGE, and NF-κB in CAL group were significantly higher than that in NCAL group, which indicate that it is involved in the occurrence and development of KD coronary injury. The expression of HMGB1, RAGE, and NF-κB were significantly elevated in the coronary artery of young rabbits with KD, and its expression was localized in endothelial cells, which may play an important role in the occurrence of KD coronary artery lesion, while its exact mechanisms of action warrant further investigation.

## Data Availability

All data generated or analyzed during this study are included in this published article.

## Abbreviations

KD: Kawasaki disease
CAL: Coronary artery lesions
NCAL: No coronary artery lesions
HMGB1: High-mobility group box-1 protein
RAGE: Receptor for advanced glycation end-products
NF-κB: Nuclear factor kappa-B
IVIG: Intravenous immunoglobulin
RT-qPCR: Reverse transcription-quantitative polymerase chain reaction
H&E: Hematoxylin–eosin
ELISA: Enzyme-linked immunosorbent assay
PBMC: Peripheral blood mononuclear cell
MAPK: Mitogen-activated protein kinase
ARDS: Acute respiratory distress syndrome
SAPK: Stress-activated protein kinase
JNK: c-Jun N-terminal kinase
BSA: Bovine serum albumin
ERK: Extracellular regulated protein kinases
OCT: Optimal cutting temperature compound
DAB: Diaminobenzidine
RNA: Ribonucleic acid
cDNA: Complementary DNA
SDS-PAGE: Sodium dodecylsulphate polyacrylamide gel electrophoresis
PVDF: Polyvinylidene fluoride
TLR: Toll-like receptor
